# Ototoxicity of cypermethrin in Wistar rats^☆^

**DOI:** 10.1016/j.bjorl.2019.02.007

**Published:** 2019-04-30

**Authors:** Eduarda Oliveira Cunha, Aléxia dos Reis, Mateus Belmonte Macedo, Márcia Salgado Machado, Eliane Dallegrave

**Affiliations:** a Universidade Federal de Ciências da Saúde de Porto Alegre, Programa de Pós-graduação em Patologia, Porto Alegre , RS , Brazil; b Universidade Federal de Ciências da Saúde de Porto Alegre, Departamento de Fonoaudiologia, Porto Alegre, RS, Brazil; c Universidade Federal de Ciências da Saúde de Porto Alegre, Departamento de Farmacologia, Programa de Pós-graduação em Patologia, Porto Alegre, RS, Brazil

**Keywords:** Ototoxicity, Cypermethrin, Rats, Ototoxicidade, Cipermetrina, Ratos

## Abstract

**Introduction:**

This study presents the effect of cypermethrin on the cochlear function in Wistar rats post-subchronic inhalation exposure. Worldwide several pesticides are described as causing health disorders. Cypermethrin is currently one of the most commonly used, however, little is known about its harmful effects, especially related to hearing. Human studies have associated pesticides with hearing disorders, but they present limited conclusions due to the multiple factors to which the population is exposed, such as noise.

**Objective:**

Mimic human exposure to cypermethrin and to verify the effects on cochlear function.

**Methods:**

It is a subchronic inhalation animal study (6 weeks, 4 hours/day), using 36 male Wistar aged 60 day. Rats were randomly assigned into three groups: Control (12 rats exposed to inhalation of water); Positive Control for auditory lesion (12 rats administrated with 24 mg/kg intraperitoneal cisplatin); Experimental (12 rats exposed to inhalation of cypermethrin – 0.25 mg/L). Animals were evaluated by distortion product otoacoustic emissions pre- and post-exposure.

**Results:**

The frequencies of 8, 10 and 12 kHz in both ears (right *p* = 0.003; 0.004; 0.008 and left 0.003; 0.016; 0.005 respectively) and at frequencies 4 and 6 in the right ear (*p* = 0.007 and 0.015, respectively) in the animals exposed to cypermethrin resulted in reduction.

**Conclusion:**

Subchronic inhalation exposure to cypermethrin provided ototoxicity in rats.

## Resumo

**Introdução:**

Este estudo apresenta o efeito da cipermetrina sobre a função coclear em ratos Wistar após exposição por inalação subcrônica. Em todo o mundo, vários pesticidas são descritos como causadores de distúrbios de saúde. A cipermetrina é atualmente um dos mais utilizados, porém pouco se conhece sobre seus efeitos deletérios, principalmente relacionados à audição. Estudos em humanos associaram pesticidas a alterações auditivas, mas apresentaram conclusões limitadas devido aos múltiplos fatores aos quais a população está exposta, como, por exemplo, o ruído.

**Objetivo:**

Mimetizar a exposição humana à cipermetrina e verificar os seus efeitos na função coclear.

**Método:**

Estudo de inalação subcrônica em animais (6 semanas, 4 horas/dia), 36 ratos machos Wistar com 60 dias. Os ratos foram distribuídos aleatoriamente em três grupos: controle (12 ratos expostos à inalação de água); controle positivo para lesão auditiva (12 ratos com administração de 24 mg/kg de cisplatina intraperitoneal); e experimental (12 ratos expostos a inalação de cipermetrina – 0,25 mg/L). Os animais foram avaliados por emissões otoacústicas por produto de distorção, pré e pós-exposição.

**Resultados:**

As frequências de 8, 10 e 12 kHz em ambas as orelhas (direita *p* = 0,003; 0,004; 0,008 e esquerda 0,003; 0,016; 0,005 respectivamente) e frequências 4 e 6 na orelha direita (*p* = 0,007 e 0,015, respectivamente) apresentaram redução nos animais expostos à cipermetrina.

**Conclusão:**

A exposição subcrônica por inalação à cipermetrina resultou em ototoxicidade em ratos.

## Introduction

Cypermethrin (*RS*)-α-cyano-3-phenoxybenzyl(1*RS*,3*RS*;1*RS*,3*SR*)-3-(2,2-dichlorovinyl)-2,2-dimethylcyclopropanecarboxylate) is a synthetic pyrethroid substance for agricultural, animal production, public health campaigns for vector control, as well as for domestic use.^1^, ^2^ This substance shows a high photostability, a non-persistence in the environment and less toxicity for human use, which makes it widely applicable.^2^, ^3^ Recently, cypermethrin has become one of the dominant pesticides, replacing carbamate and organophosphates that are considered more toxic.^4^

According to the World Health Organization (WHO), there are more than 18.2 per 100,000 cases of acute pesticide poisoning in agricultural workers per year, and probably the subacute cases are worse, but there are difficulties in documenting these data. Furthermore, not all pesticide effects are known. In fact, there is a concern that different chemical substances may cause hearing damage, such as the pesticides.^5^, ^6^, ^7^

Human studies indicate that exposure to pesticides may cause damage to the auditory system.^6^, ^8^, ^9^, ^10^, ^11^ However, these researches have limitations and there is a lack of studies evaluating ototoxicity due to exposure to pesticides. In animal models, different modalities of administration of the substance are used. Often the modality of exposure is not correlated with occupational exposure and anesthesia was used for auditory evaluation.^8^ Moreover, most of the researches that related pesticides with hearing loss are case reports or human studies exposed to noise machinery and to other factors that also can cause hearing damage.^8^, ^10^, ^12^, ^13^, ^14^, ^15^, ^16^, ^17^, ^18^

An experimental study with organophosphates in guinea pigs^14^ has shown cochlear morphological changes, with lesions in the outer hair cells concomitantly with the presence of otoacoustic emissions.^14^ However, the relation of pesticides with hearing damage is still unclear because of the multiple factors to which humans are exposed. Therefore, our study in animal models exposed only to cypermethrin, free from the bias of noise, allows a characterization of the implications of a pesticide on the auditory system. Thus, this study was aimed to evaluate the ototoxic effects of subchronic inhalation of cypermethrin in Wistar rats through distortion product otoacoustic emissions.

## Methods

This was an experimental study based on subchronic inhalation toxicity test, number 413, from the Organization for Economic Co-operation and Development (OECD) Guidelines for the Testing of Chemicals.^19^ All procedures involving animals were approved by the Ethics Committee on Animal Use under n 323/15.

### Animals

Thirty six male Wistar rats (*Rattus norvegicus*) aged 60 days were used in the experiments (body weight approximately 300 ± 50 g). The animals were kept under controlled conditions of the bioterium, 12 h light/dark cycle, receiving water and food ad libitum, except during exposure. Only animals with no signs of external ear pathology and Distortion Product Otoacoustic Emissions (DPOAE) present in all tested frequencies (4, 6, 8, 10 and 12 kHz) were selected for the experiment. The rats were randomly assigned to three groups: control (*n* = 12), positive control (*n* = 12) and experimental (*n* = 12). All animals were exposed to the same background noise level which was kept below 60 dB at all times.

### Substances

The commercially available formulation of Cypermethrin (type II pyrethroid) was dissolved in distilled water at the concentration of 0.25 mg/L (1/10 of rat inhalation LC_50_). Cisplatin, an anti-neoplastic medicine, was used as a positive control for ototoxicity, totaling 24 mg/kg. Each animal received 8 mg/kg intraperitoneally, per day, diluted in 10 mL/kg of saline solution.

### Distortion product otoacoustic emissions

DPOAEs were recorded using the OtoreadClinical®, Interacoustics®. Two tones (f1 and f2) were used as acoustic stimuli (f1/f2, f2:f1 ratio fixed at 1.22). The f1 and f2 tones were presented at a stimulus level of 65 and 55 dB SPL (Sound Pressure Level). DPOAE tests were performed with an infant size hearing probe placed into the external ear canal of the rat and tested at frequencies of 4, 6, 8, 10 and 12 without use of anesthesia. Distortion product otoacoustic emissions were performed in the control and experimental groups before (pre-exposure) and after (post-exposure) the exposure period (0 and 42 days), and in the positive control previously the 1st administration and 24 h after the 3rd administration.

### Exposures

In our protocol of inhalation exposure, we used chambers of 56 l. The chambers were joined to ultrasonic nebulizers (avoiding noise) as an inlet stream, and to an exhaust system. This system was created avoiding noise exposure by an enclosure for acoustic insulation.

The animals were adapted to our protocol during a period (5 days). They were trained to habituation, firstly with the probe used in the auditory evaluation and with the sound that occurs during the DPOAE, as well as adaptation in the exposure chamber, initially for 1 h (day 1), the 2nd day for 2 h, the third day for 3 h and on the 4th day for 4 h (using only air flow). On day 5 air flow was used with water vapor over 4 h.

The control group was exposed to water (vehicle for dilution of the formulation) inhalation for 4 h, 5 times a week, for 6 weeks. The experimental group was exposed to cypermethrin inhalation at a concentration of 0.25 mg/L, for 4 h, 5 times a week, for 6 weeks. This protocol was created and used by our group in inhalation exposition of pesticides.

The positive control group of 12 rats treated with 8 mg/kg cisplatin intraperitoneally, once daily, for 3 consecutive days (totaling 24 mg/kg).^8^

### Statistics analyses

Statistical analyses were performed using the SPSS statistical software package (SPSS version 21.0 for Windows, SPSS Inc., Chicago, IL, USA). Numeric variables were specified as median (25th percentile–75th percentile). The Mann–Whitney *U* test was used to compare the groups (post-exposure assessment in relation to the pre-exposure of each group) and Kruskal–Wallis (comparison of the variations post minus pre evaluation between groups). Parametric data were presented as mean and standard error of the mean and those variables that had normal distribution were evaluated by *t*-Student (relative body mass, relative organ mass). The statistical significance value was regarded as *p* < 0.05.

## Results

The effect of cypermethrin upon DPOAEs was found to be essentially the same across this frequency range of 4, 6, 8, 10 and 12 kHz.

Exposure to cypermethrin demonstrated significant reduction of DPOAE (*p* < 0.05, Mann–Whitney test) compared to pre-exposure measurements (Table 1) at frequencies 8, 10 and 12 kHz in both ears (right *p* = 0.003; 0.004; 0.008 and left 0.003; 0.016; 0.005, respectively) and at frequencies 4 and 6 in the right ear (*p* = 0.007 and 0.015, respectively).

**Table 1:** Median and interquartile ranges of DPOAE amplitudes pre- and post-subchronic inhalation exposure to cypermethrin (experimental group).

Ear	Frequency (kHz)	Pre-exposure median (quartile25/quartile75)	Post-exposure median (quartile25/quartile75)	*p*-Value (Mann–Whitney)
Right	4	9.50 (3.50/17.25)	−2.50 (−9.25/1.00)	0.007^a^
Right	6	27.50 (6.50/38.50)	0.50 (−10.75/10.25)	0.015^a^
Right	8	31.50 (16.75/46.25)	−2.00 (−25.00/14.75)	0.003^a^
Right	10	27.50 (2.00/42.25)	0.00 (−6.50/6.75)	0.004^a^
Right	12	10.00 (7.00/44.00)	0.50 (−11.50/5.25)	0.008^a^
Left	4	6.50 (4.50/14.00)	4.00 (−2.75/9.00)	0.146
Left	6	8.00 (00.00/23.00)	1.00 (−3.75/3.75)	0.077
Left	8	9.50 (7.00/33.25)	17.00 (−25.00/−1.75)	0.003^a^
Left	10	10.50 (5.00/29.50)	−0.50 (−14.50/7.25)	0.016^a^
Left	12	29.00 (10.75/34.00)	6.00 (2.50/9.00)	0.005^a^

a *p* < 0.05 (Mann–Whitney).

The same significantly reduced DPOAE was found in the cisplatin group (Table 2) at frequencies 12 kHz (*p* = 0.006) in the right ear and 4 kHz in the left ear (*p* = 0.032). The control group showed no significant difference (*p* > 0.05; Mann–Whitney test) in post-exposure measurements (Table 3), confirming the standardization of the experimental model.

**Table 2:** Median and interquartile ranges of DPOAE amplitudes pre- and post-treatment with cisplatin (positive control).

Ear	Frequency (KHz)	Pre-exposure median (quartile25/quartile75)	Post-exposure median (quartile25/quartile75)	*p*-Value (Mann Whitney)
Right	4	7.00 (4.00/12.25)	4.00 (−2.50/8.00)	0.116
Right	6	11.50 (3.50/23.50)	3.00 (0.00/7.25)	0.131
Right	8	28.00 (11.50/35.00)	18.00 (7.00/33.50)	0.195
Right	10	24.00 (12.50/34.25)	7.50 (3.25/28.00)	0.050
Right	12	24.00 (17.25/36.00)	7.00 (1.25/22.25)	0.006^a^
Left	4	6.50 (2.25/9.25)	−0.50 (−2.25/3.25)	0.032^a^
Left	6	14.00 (10.75/20.50)	7.00 (1.50/14.00)	0.136
Left	8	23.50 (18.00/28.25)	16.00 (2.00/29.25)	0.195
Left	10	25.50 (16.75/30.25)	12.00 (5.50/29.50)	0.147
Left	12	20.50 (8.00/38.50)	8.00 (4.00/26.00)	0.239

a *p* < 0.05 (Mann–Whitney).

**Table 3:** Median and interquartile ranges of DPOAE amplitudes pre and post subchronic inhalation exposure to water (control).

Ear	Frequency (kHz)	Pre-exposure median (quartile25/quartile75)	Post-exposure median (quartile25/quartile75)	*p*-Value (Mann–Whitney)
Right	4	6.50 (2.25/13.00)	10.50 (7.50/14.50)	0.255
Right	6	27.50 (15.25/33.25)	33.50 (15.00/36.25)	0.646
Right	8	28.00 (10.50/35.25)	36.50 (21.50/39.00)	0.505
Right	10	32.00 (14.00/38.00)	38.00 (34.50/43.25)	0.308
Right	12	29.50 (16.25/39.25)	42.00 (21.75/48.00)	0.195
Left	4	2.00 (0.00/12.50)	11.00 (3.75/19.50)	0.266
Left	6	16.50 (09.75/26.75)	26.00 (24.00/32.75)	0.209
Left	8	19.00 (4.25/37.50)	38.00 (29.25/39.75)	0.153
Left	10	29.00 (19.00/35.25)	30.50 (24.50/39.50)	0.724
Left	12	31.50 (25.50/39.75)	42.50 (26.50/45.50)	0.213

^a^*p* < 0.05 (Mann–Whitney).

**Figure 1:**
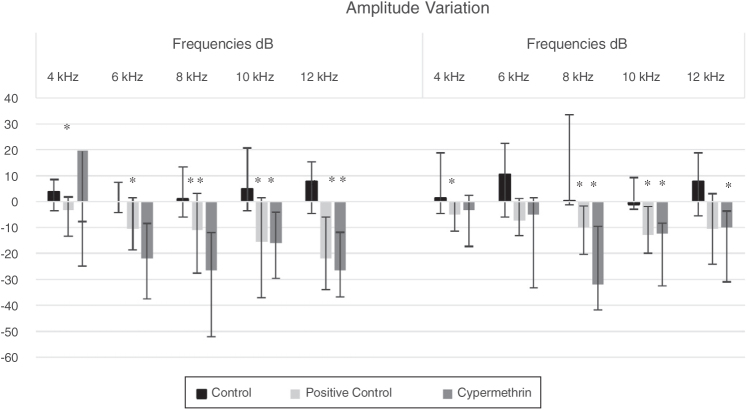
Amplitude variation of DPOAE post menus pre subchronic inhalation exposure to cypermethrin or water and post cisplatin treatment. * *p* < 0.05 (Kruskal–Wallis).

The groups showed statistical difference (*p* < 0.05; Kruskal–Wallis) in relation to the variation of the medians post-exposure menus pre-exposure (Fig. 1).

There were significant differences among groups in the right ear at frequencies of 4, 6, 8 kHz (cypermethrin versus control) and in the 12 kHz (cypermethrin and cisplatin was different from control group; *p* < 0.005; Kruskal–Wallis). In the left ear, there was a significant difference among groups at the frequencies of 8 and 10 kHz (cypermethrin and cisplatin was different from control group; *p* < 0.005; Kruskal–Wallis). In all cases, the changes observed in the animals exposed to cypermethrin were similar to the cisplatin group, considered an ototoxic substance.

The relative gain of body mass (mean ± standard error) in the period was similar (*p* = 0.270; *t*-Student test) between the control (145.33 ± 5.91%) and cypermethrin (156.00 ± 6.75%).

Therefore, most of the animals demonstrated transient clinical signs such as piloerection, dyspnea and pruritus. The clinical signs were more evident at the beginning of the exposure (between 30 and 240 minutes) and in the first two weeks.

We did not found macroscopic alterations in the organs of the animals used in the experimental protocol.

## Discussion

In our study, we demonstrated ototoxicity by subchronic inhalation exposure to cypermethrin in Wistar rats. The results of cypermethrin exposure demonstrated reduction in amplitude on DPOAE.

Cypermethrin is considered to produce lower toxic than other pesticides, even though it is known as a neurotoxic substance which can also affect auditory system.^16^, ^17^, ^18^ Studies have demonstrated the neurologic impact of cypermethrin in animal models. Rats have shown tremors, seizures, writhing, and salivation as well as burrowing behavior following cypermethrin exposure in low doses.^18^, ^20^

In human population, cypermethrin is more associated with acute poison and allergic or respiratory effects.^3^

Researchers have pointed out the hearing impact of humans exposed to pesticides, although most of these studies were done with agricultural workers who are mostly exposed to more than one type of pesticide and to other causes of hearing loss.^6^, ^8^ Moreover, some studies have reported association between organophosphate exposure and reduction of the hearing, but no one of these studies have single substance exposure such as cypermethrin.^8^, ^9^, ^10^, ^11^, ^21^

The literature suggests that pesticides exposure can damage the auditory system, however in human studies different factors may influence the outcome.^8^ In addition, some studies do not have exposure modality and duration precisely defined.^8^, ^10^, ^21^, ^22^ No one of these articles described in the literature has performed an inhalation exposure as our study have done to evaluate cochlear function.^8^, ^10^

In this study, we have used the DPOAE to evaluate the impact on cochlear function. DPOAE is widely used as a screening method to evaluate newborns in many countries.^23^ In some studies, DPOAE is used as a screening device for ototoxic substances, because it allows us evaluate the high frequencies, as well as the first frequencies that most ototoxic substances affected. We demonstrated that this test can be performed without anesthesia once rats are trained.^6^, ^10^, ^24^ Then, the decrease of amplitude by cypermethrin exposure observed in our study can be directly correlated.

To the best of our knowledge, this is the first time that the exposure to cypermethrin was evaluated without anesthesia. On the contrary of previous studies that have used pesticides^7^, ^8^, ^10^, ^11^, ^14^ considered more toxic than cypermethrin, we mimiced the human exposure. Some anesthetics may interfere with the results of studies, changing the latency and amplitude of the waves.^5^, ^24^ In this study, it was possible to evaluate the animals without anesthesia, due to the previous acclimatization to the instruments.

Moreover, ototoxicity is described as a reversible or irreversible damage of inner ear functions due to the exposure to chemical substances.^22^, ^25^ The ototoxicity caused by cypermethrin could impact the quality of life of agricultural workers who are routinely exposed. As a result of ototoxicity in humans, Cypermethrin is referred to in literature as a low systemic toxic agent, and in our study that no systemic alterations were observed.

In this paper we have assessed the effects on cochlear function of subchronic inhalation to cypermethrin in Wistar rats, and developed an animal protocol that eliminated the confounders that are associated with hearing loss in human research. Furthermore, our findings were consistent, since all control groups (positive control and control) performed as the reported in the literature. The animals manifested few clinical signs, which indicate that ototoxicity associated with cypermethrin exposure was experienced even without systemic toxicity.

There were limitations to this study based on the difficulty explaining the mechanism of damage on cochlear function, as well as in the inner cells. We believe that cypermethrin can damage other structures. For future studies, we suggest the histopathological analysis of inner cells as well as evaluation of the central auditory system.

Ototoxicity should be considered during the diagnosis of hearing loss, especially in agricultural workers or others exposed to pesticides. These individuals are exposed to multiple factors and represent a vulnerable population to hearing loss. In clinical practice, we may consider the association of experimental and human studies due to the importance of understanding the mechanism of the substances to which our patients are exposed. Most of studies about ototoxicity in PubMed are related to use of certain medicines, such as cisplatin and antibiotics.^7^

Even though this is an experimental study, due to the absence of literature associating the use of drugs and exposure to chemical substances with a hearing effects, it is necessary to correlate clinical studies with experimental studies.

## Conclusion

This study provides evidences that subchronic inhalation exposure to cypermethrin at low concentrations induced ototoxicity in rats.

## Conflict of interest

The authors declare no conflicts of interest.
